# Integrative single-cell and genomic analysis reveals NMB as a driver of metastatic adaptation in esophageal squamous cell carcinoma via metabolic rewiring and immune evasion

**DOI:** 10.3389/fcell.2026.1914292

**Published:** 2026-08-31

**Authors:** Zhikai Cao, Dongchen Tian, Long He, Jiahui Zhang, Jing Zeng, Yao Tian, Xiaofeng Liu, Yingxi Li

**Affiliations:** 1 Department of Thoracic Surgery, The Affiliated LiHuiLi Hospital of Ningbo University, Ningbo, Zhejiang, China; 2 Health Science Center, Ningbo University, Ningbo, Zhejiang, China; 3 The First Department of Breast Cancer, Key Laboratory of Cancer Prevention and Therapy, Tianjin’s Clinical Research Center for Cancer, National Clinical Research Center for Cancer, Key Laboratory of Breast Cancer Prevention and Therapy, Tianjin Medical University Cancer Institute and Hospital, Tianjin Medical University, Tianjin, China; 4 Department of Thyroid and Breast Surgery, Bishan Hospital of Chongqing Medical University, Chongqing, China

**Keywords:** esophageal squamous cell carcinoma, genomic instability, NMB, single-cell transcriptomics, somatic mutation

## Abstract

**Background:**

Esophageal squamous cell carcinoma (ESCC) has high mortality, and metastasis is the leading cause of patient death. Neuromedin B (NMB) promotes tumor development in various cancers, yet its role in ESCC metastasis remains unclear.

**Methods:**

We integrated single-cell transcriptomic data from matched primary and metastatic ESCC lesions (GSE309392) with bulk transcriptomic cohorts from TCGA and GSE53624. In silico gene perturbation, ligand-receptor communication analysis, and single-cell prognostic model construction were performed, followed by functional validation through siRNA-mediated NMB knockdown in TE-1 and KYSE30 cell lines.

**Results:**

NMB was identified as a key gene enriched in metastatic ESCC lesions, and its high expression was associated with coordinated upregulation of oxidative phosphorylation pathway genes and aldo-keto reductase family antioxidant enzymes (AKR1C1, AKR1C2, AKR1B10). Genomic analysis revealed that NMB-high tumors carried a higher clonal mutation burden and a markedly increased frequency of NFE2L2 activating mutations (23% vs*.* 8%, *P* = 0.04). In silico knockout and correlation analysis identified AKR1C1 as a downstream effector of NMB. NMB expression was negatively correlated with CD8^+^ T cell and activated NK cell infiltration. CellChat analysis revealed communication between NMB-positive cells and monocytes via the TGM2–ADGRG1 axis, and specifically detected IFNG signaling. In the single-cell prognostic model, NMB-positive cells accounted for 50% of the high-risk group but only 20% of the low-risk group. TCGA-based survival analysis demonstrated that high NMB expression was associated with shorter overall survival (HR = 2.98, *P* = 0.03). *In vitro* NMB-targeted RNA interference markedly inhibited proliferation, colony formation, and migration in TE-1 and KYSE30 cells. CMap screening identified the endothelin-PDE5-cGMP axis as a potential therapeutic target.

**Conclusion:**

NMB serves as a key driver of metastatic adaptation in ESCC, conferring a survival advantage to tumor cells during metastatic colonization through genomic evolution and immune remodeling, with metabolic adaptation as a downstream consequence of genomic alterations.

## 1 Introduction

Esophageal squamous cell carcinoma (ESCC) is the predominant histological subtype of esophageal cancer, accounting for approximately 85% of global cases (Zhang et al., 2026). Esophageal squamous cell carcinoma (ESCC) has an extremely poor prognosis, with fewer than 20% of patients surviving for more than 5 years after diagnosis (Thrift, 2021). Malignant metastasis is the leading cause of poor prognosis and treatment failure in patients with ESCC. The metastatic progression of ESCC is accompanied by aggravated tumor cellular heterogeneity and dynamic remodeling of the tumor microenvironment (Sheng et al., 2025; Zheng et al., 2025), and genomic instability serves as the fundamental driver of such high tumor heterogeneity.

Genomic mutation disorder is a core molecular hallmark of ESCC. Whole-genome sequencing reveals extensive copy number variations in ESCC, with the TP53 mutation rate ranging from 60% to 90% (Efe et al., 2023). Recurrent mutations are also commonly found in key pathway genes such as NOTCH1, NFE2L2 and CDKN2A (Lu et al., 2025; Cui et al., 2020; Liu et al., 2025; Yin et al., 2024). In addition, diverse genomic mutations can induce the generation of tumor-specific neoantigens, regulate the expression of immune checkpoint molecules including programmed death ligand 1 (PD-L1) (Li et al., 2025), and remodel the tumor immune microenvironment (TME) (Zhang et al., 2025). TME is a critical factor driving distant metastasis in ESCC (Xia et al., 2025; Boyle and Lei, 2023; Wu et al., 2021). Within the TME, abundant infiltration of immunosuppressive cells, including regulatory T cells (Tregs), myeloid-derived suppressor cells, and M2-polarized tumor-associated macrophages, profoundly suppresses the antitumor immune functions of CD8^+^ T lymphocytes and natural killer (NK) cells, thereby creating a permissive niche for tumor immune evasion and subsequent distant colonization (Jin et al., 2025; Yin et al., 2025; Zhao et al., 2026; Cheng et al., 2026). Although the immunosuppressive TME is known to drive ESCC metastasis, its upstream regulators remain unclear. Thus, we sought to identify potential molecular targets in ESCC related to genomic alterations and tumor microenvironment.

Neuromedin B (NMB), a secreted bioactive polypeptide belonging to the bombesin-like peptide family, exerts biological functions by specifically binding to its G-protein coupled receptor NMBR (Xia et al., 2025; Boyle and Lei, 2023; Wu et al., 2021). Many studies have verified the oncogenic role of NMB in multiple solid tumors such as cervical cancer, adenocorticotropic hormone adenoma and colorectal cancer (Gao et al., 2024; Fan et al., 2025; Yu et al., 2026). NMB facilitates tumor cell proliferation and perineural invasion, and induces the formation of an immunosuppressive tumor microenvironment. Despite these findings, the role of NMB in ESCC remains poorly understood. The cellular distribution of NMB, as well as its correlations with genomic mutations, cellular origin, and tumor functional heterogeneity, has not been clarified. The specific molecular mechanisms underlying its regulatory effects on tumor progression also require further exploration. Our research further examined the correlations between NMB expression, genomic instability and immune microenvironment remodeling. These findings position NMB as a candidate molecular subtyping marker and provide a rationale for its evaluation as a therapeutic target in ESCC.

## 2 Materials and methods

### 2.1 Data sources

Single-cell transcriptomic data were obtained from the GEO database under accession GSE309392, comprising 6 ESCC samples including 3 primary tumors and pulmonary metastatic lesions. Bulk transcriptomic data included two independent cohorts (Zhang et al., 2026): the GSE53624 cohort from GEO, containing ESCC tumor tissues with paired adjacent normal tissues and corresponding clinical follow-up information; and (Thrift, 2021) the TCGA-ESCA cohort, containing transcriptomic sequencing data from tumor and normal tissues with corresponding clinical survival data. Somatic mutation data for ESCC patients were downloaded from the TCGA database for mutational profile analysis.

### 2.2 Single-cell data preprocessing and batch correction

Single-cell data preprocessing was performed using the R package Seurat (v4.3.0). Expression matrices from each sample were read and merged into a single Seurat object with sample grouping information added. Quality control filtering criteria were: gene count 200–6,000 and mitochondrial gene percentage <20%. Doublets were identified and removed using the scDblFinder algorithm, followed by exclusion of mitochondrial and ribosomal genes. Post-quality control data were normalized using LogNormalize, and 2000 highly variable genes were selected. After ScaleData scaling, PCA dimensionality reduction was performed. Batch effects were corrected using the Harmony algorithm with sample ID as the grouping variable. The first 30 principal components were selected for nearest neighbor computation, with resolution set to 0.3 for cell clustering, followed by nonlinear dimensionality reduction for visualization.

### 2.3 Cell type annotation

Cell types were manually annotated based on expression profiles of canonical marker genes: epithelial cells (EPCAM, KRT19, CDH1); T/NK cells (CD3D, CD8A, NKG7); monocytes (FCN1, CD14, C1QA); B/plasma cells (JCHAIN, MS4A1, CD79A); mast cells (MS4A2, TPSAB1, CPA3); fibroblasts (COL1A1, DCN, PDGFRB); endothelial cells (PECAM1, CDH5, VWF); and dendritic cells (CD1C, ITGAX, CD83). Cell type annotations were stored in metadata.

### 2.4 Malignant cell identification and subpopulation analysis

Copy number variation analysis was performed on a per-sample basis using the inferCNV (Chen et al., 2022) package, with T/NK cells as normal reference cells. CNV scores and CNV correlation coefficients were calculated for each epithelial cell. Thresholds of CNV score >0.001 and correlation coefficient >0.4 were applied to distinguish malignant from normal epithelial cells. All malignant epithelial cells were extracted for re-clustering. After PCA dimensionality reduction, the DBSCAN algorithm was applied to identify and remove outlier cells in UMAP space, yielding refined malignant cell subpopulations.

### 2.5 Trajectory analysis of metastatic malignant cells

Differentiation trajectories of malignant epithelial cells were constructed using the Slingshot algorithm based on UMAP dimensionality reduction results. Dynamically expressed genes during differentiation were identified based on pseudotime values (n_candidates = 200), clustered by expression patterns, and subjected to GO-BP functional enrichment analysis. Pseudotime heatmaps were generated to display gene expression dynamics.

### 2.6 Transcription factor regulatory network analysis

Transcription factor (TF) activity in malignant cells was quantitatively analyzed using the decoupleR (Badia et al., 2022) package combined with the CollecTRI transcription factor regulatory network and the ULM algorithm. Single-cell normalized expression matrices were extracted and imported with regulatory files. The ULM algorithm was applied to calculate TF activity scores for each transcription factor in each cell, generating a TF activity expression matrix for subsequent analysis. Clustering heatmaps were generated to display transcriptional regulatory characteristics across subpopulations.

### 2.7 Bulk transcriptomic data integration and differential expression analysis

The GSE53624 and TCGA-ESCA cohorts were independently preprocessed. GSE53624 data were obtained from GEO with gene symbol annotation using the provided probe annotation file; for multiple probes mapping to the same gene, the median expression was taken. TCGA data were downloaded from GDC as STAR-Count files. Common genes between the two cohorts were extracted, and expression values were log2 (x+1) transformed followed by ComBat batch correction with data source as the batch variable to eliminate inter-cohort technical variation. Linear models were constructed using the limma package for differential expression analysis between tumor and normal tissues, with |log2FC|>1 and adjusted *P* < 0.05 as thresholds for significant differential genes. Volcano plots were generated to display the overall differential distribution. Venn diagrams were generated using the online tool Bioinformatics (http://www.bioinformatics.com.cn). Correlations between gene expression levels were assessed using the Pearson correlation coefficient.

### 2.8 Genomic mutation profile analysis

TCGA-ESCC somatic mutation data were processed using the Maftools (Mayakonda et al., 2018) package. Samples were divided into high and low NMB expression groups based on the median NMB expression level. Clonal mutation proportions (defined as VAF>0.3), total mutation burden (TMB), and driver gene mutation frequencies were compared between the two groups. Copy number variation characteristics and mutational signature profiles were also analyzed.

### 2.9 Survival analysis and SIDISH prognostic model construction

The optimal NMB expression cutoff was determined by surv_cutpoint (minprop = 0.1) using GSE53624 follow-up data. Kaplan-Meier curves and log-rank tests compared overall survival between high and low NMB groups, and Cox models yielded HRs with 95% CIs. ComBat-corrected TCGA-ESCA and GSE53624 datasets were integrated with survival data for combined survival validation. The Python-based SIDISH algorithm (Jolasun et al., 2025) built a single-cell prognostic risk model. Normalized malignant single-cell expression matrices were used for training, with bulk transcriptomes and survival data as inputs. Data alignment and integration were completed via SIDISH’s built-in preprocessing module. Joint training of deep VAE and DeepCox extracted malignant cell gene features and mapped them to bulk data. LASSO-Cox regression screened prognostic genes and generated a risk score formula. Samples were split into high/low-risk subgroups by median risk score, and NMB expression distribution among subgroups was analyzed to evaluate combined prognostic stratification performance.

### 2.10 Immune infiltration analysis

The CIBERSORT, xCell, and MCP-counter algorithms were applied to estimate immune cell infiltration proportions, and ESTIMATE was used to calculate ImmuneScore, StromalScore, and tumor purity. Pearson correlation coefficients between NMB expression and these parameters were calculated, with P < 0.05 as significant.

### 2.11 In silico gene knockout analysis

Based on single-cell transcriptomic data, malignant cells were first selected from the overall cell population, and NMB-positive (NMB+) malignant cells were further selected using NMB expression > 0 as the threshold for subsequent analysis. Cell count matrices were extracted, and the top 2000 most variable genes were selected to construct the input matrix. Single-cell-level *in silico* knockout perturbation analysis of the NMB gene was performed using the scTenifoldKnk package. Parameters were set as follows: quality control enabled (qc = TRUE), mitochondrial gene ratio threshold 0.1 (qc_mtThreshold = 0.1), minimum gene detection threshold 1,000 (qc_minLSize = 1,000), number of subnetworks 10 (nc_nNet = 10), and sampling cell number 500 (nc_nCells = 500). Differentially regulated genes after knockout were extracted, with adjusted P < 0.05 as the significance threshold. Volcano plots and bar plots were generated using ggplot2. KEGG pathway and GO biological process enrichment analyses were performed using the clusterProfiler package with human species background (hsa) and enrichment threshold *P* < 0.05.

### 2.12 Cell-cell communication analysis

Based on single-cell data, unknown cells were excluded, and malignant cells were divided into two groups according to NMB expression with downsampling (≤500 cells per group). Ligand-receptor communication was analyzed using CellChat v2.0 and the human database, with significant interactions filtered at *P* < 0.01. Pathway and ligand-receptor pair differences between the two groups were compared.

### 2.13 Drug sensitivity prediction

From bulk-RNA differential expression analysis, the top 50 upregulated and 50 downregulated genes were selected for drug enrichment score calculation against the CMAP (Zhang et al., 2021) database. Based on connectivity scores, the top 15 positive and negative drugs were plotted as bar charts; the top 30 drugs were used to construct a single-column heatmap; and the top 20 drugs with annotation information were used to generate OncoPrint plots for candidate drug screening and visualization.

### 2.14 Cell culture and transfection

TE-1 and KYSE30 cells were obtained from the Type Culture Collection of the Chinese Academy of Sciences and cultured in RPMI-1640 with 10% FBS at 37 °C in 5% CO_2_. Cells at 60%–70% confluence were transfected with NMB-targeting siRNAs or negative control using Lipofectamine 3,000. Medium was replaced after 6 h, and cells were harvested at 48 h post-transfection. siRNA sequences are listed in Supplementary Table S2.

### 2.15 Quantitative real-time PCR

Total RNA was extracted from transfected cells using Trizol reagent and reverse-transcribed to cDNA. Quantitative PCR was performed using SYBR Green master mix with GAPDH as internal control. Relative NMB mRNA expression was calculated using the 2^(-ΔΔCt)^ method. The sequences of primers of NMB and GAPDH are listed in Supplementary Table S1.

### 2.16 MTT proliferation assay

Transfected cells were plated in 96-well plates with 6 replicate wells per group. Following 1–5 days of culture, MTT solution was added to each well and incubated for 4 h. Formazan crystals were dissolved in DMSO, and optical density was measured at 490 nm using a microplate reader. Proliferation curves were generated.

### 2.17 Colony formation assay

Single-cell suspensions from each group were seeded at 800 cells/well in 6-well plates and cultured for 10 days. Colonies were fixed with 4% paraformaldehyde, stained with crystal violet, photographed, and counted. Each group had 3 replicate wells.

### 2.18 Transwell migration assay

Migration was assessed using Transwell chambers without Matrigel. Serum-free cell suspensions were added to the upper chamber, with 10% FBS medium in the lower chamber as chemoattractant. After 24 h of incubation, cells on the upper surface were removed, and migrated cells were fixed, stained, and counted under microscopy.

### 2.19 Statistical analysis

Data are presented as mean ± standard deviation. GraphPad Prism software was used for analysis. Comparisons among multiple groups were performed using one-way ANOVA. *P* < 0.05 was considered statistically significant.

## 3 Results

### 3.1 Single-cell transcriptomic Atlas construction and cell type identification in ESCC

**FIGURE 1:**
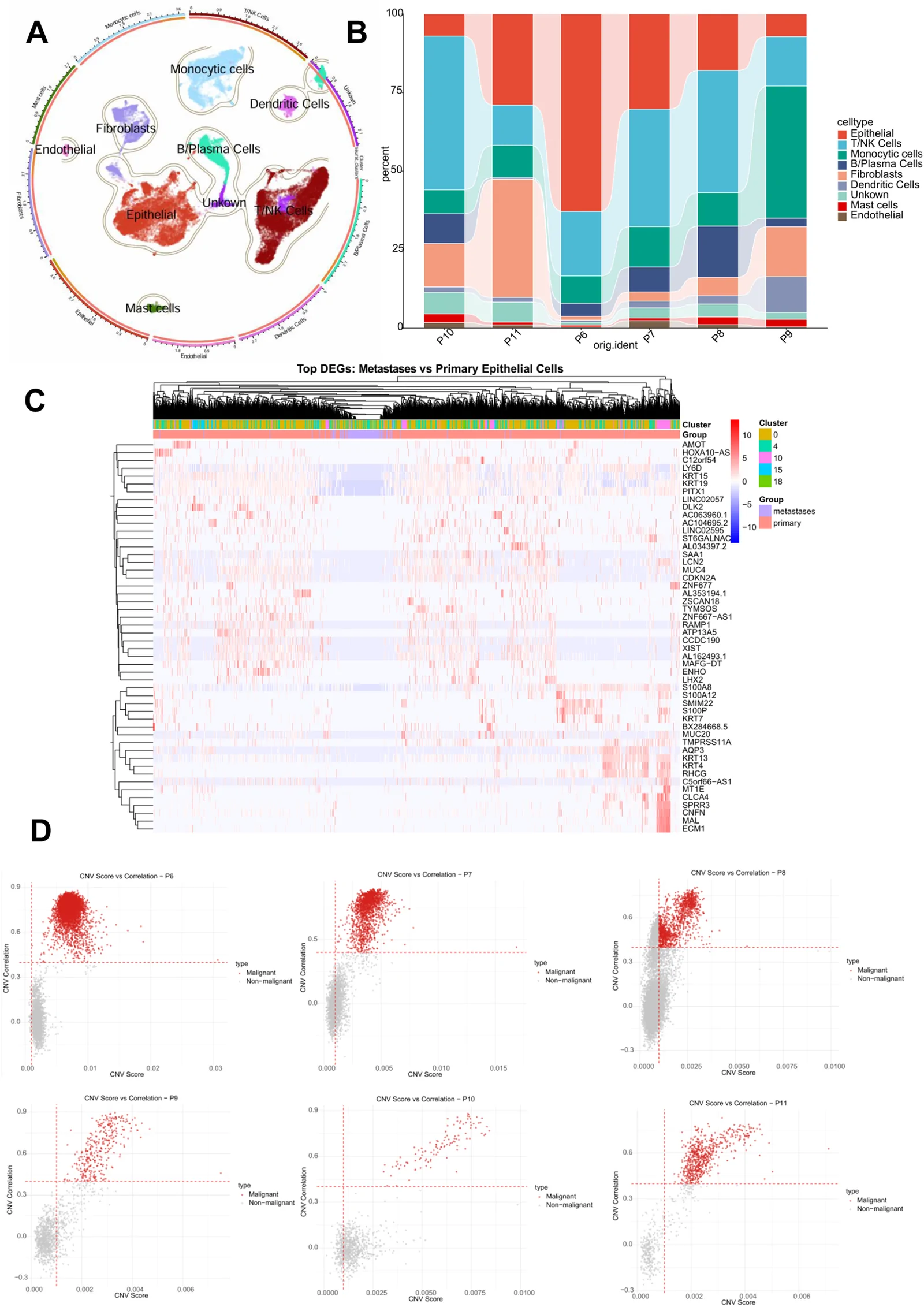
Single-Cell Transcriptomic Atlas Construction and Cell Type Identification in ESCC. **(A)** UMAP visualization of cell subpopulations based on transcriptomic features, annotated with major cell types in the tumor microenvironment. **(B)** Stacked bar chart showing the proportion of each cell type across different samples. **(C)** Heatmap of cell subpopulation marker genes, with genes as rows and cells as columns, color intensity representing normalized expression levels. **(D)** Scatter plot of CNV score versus gene expression features for distinguishing tumor from non-tumor cells, showing copy number variation characteristics and tumor progression-associated gene expression across subpopulations.

Quality control of single-cell data from 6 ESCC samples yielded high-quality cells for downstream analysis. Harmony batch correction resolved distinct clusters by cell type (Supplementary Figures S1A, B; Supplementary Figures S5F–G) Marker gene annotation defined 9 major cell types: epithelial cells, T/NK cells, monocytes, B/plasma cells, mast cells, fibroblasts, endothelial cells, dendritic cells, and an uncharacterized cluster (Figures 1A,B; Supplementary Figures S1C, D). Epithelial cells extracted from primary and metastatic lesions displayed distinct transcriptional profiles on heatmap analysis (Figure 1C; Supplementary Figures S1E, F) inferCNV malignancy assessment showed that malignant epithelial cells carried higher CNV scores compared to normal epithelial cells and immune reference cells (Figure 1D; Supplementary Figures S1G, H).

### 3.2 Identification of a metastatic tumor subpopulation with invasive malignant features

**FIGURE 2:**
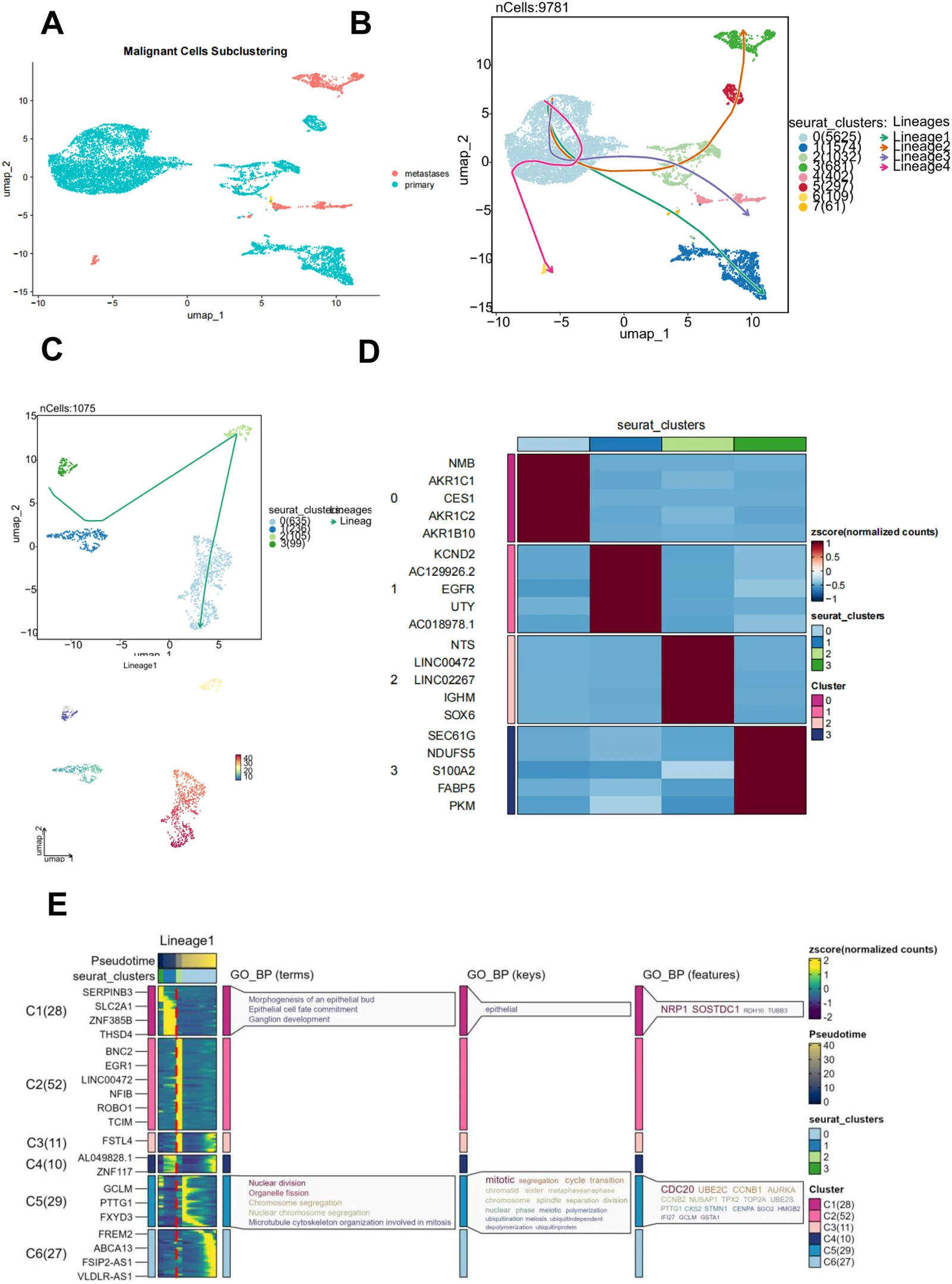
Identification of a Metastatic Tumor Subpopulation with Invasive Malignant Features. **(A)** UMAP clustering of malignant cells based on transcriptomic features, colored by primary vs. metastatic origin. **(B)** Pseudotime trajectory of all malignant cells, with different colors representing subpopulations and trajectory lines indicating evolutionary direction and branches from primary to metastatic tumors. **(C)** Pseudotime analysis of metastatic malignant cells, with upper panel colored by subpopulation and lower panel by pseudotime gradient. **(D)** Heatmap of marker gene expression across malignant subpopulations, with genes as rows and subpopulations as columns. **(E)** Pseudotime dynamic gene expression heatmap for Lineage 1, with differentially expressed genes clustered into 6 modules and corresponding GO biological process enrichment terms annotated.

To explore the evolutionary characteristics of tumor cells during metastasis, we performed re-clustering on all malignant cells and conducted trajectory inference. Three differentiation lineages were identified, which were derived from primary tumor cells (Figures 2A,B; Supplementary Figure S2B). We then extracted metastatic tumor cells for independent re-clustering (Supplementary Figure S2B), and found that these cells converged into a single differentiation lineage (Figure 2C). Within this lineage, subpopulation 0 was the evolutionary terminal state of primary tumor cells and had the highest proportion of NMB-enriched cells. These cells also exhibited high expression of antioxidant genes including AKR1C1, AKR1C2 and AKR1B10 (Figure 2D). Dynamic feature analysis further illustrated the changes in biological functions and molecular markers of tumor cells along the differentiation trajectory (Figure 2E).

### 3.3 NMB as a risk factor for ESCC metastasis

**FIGURE 3:**
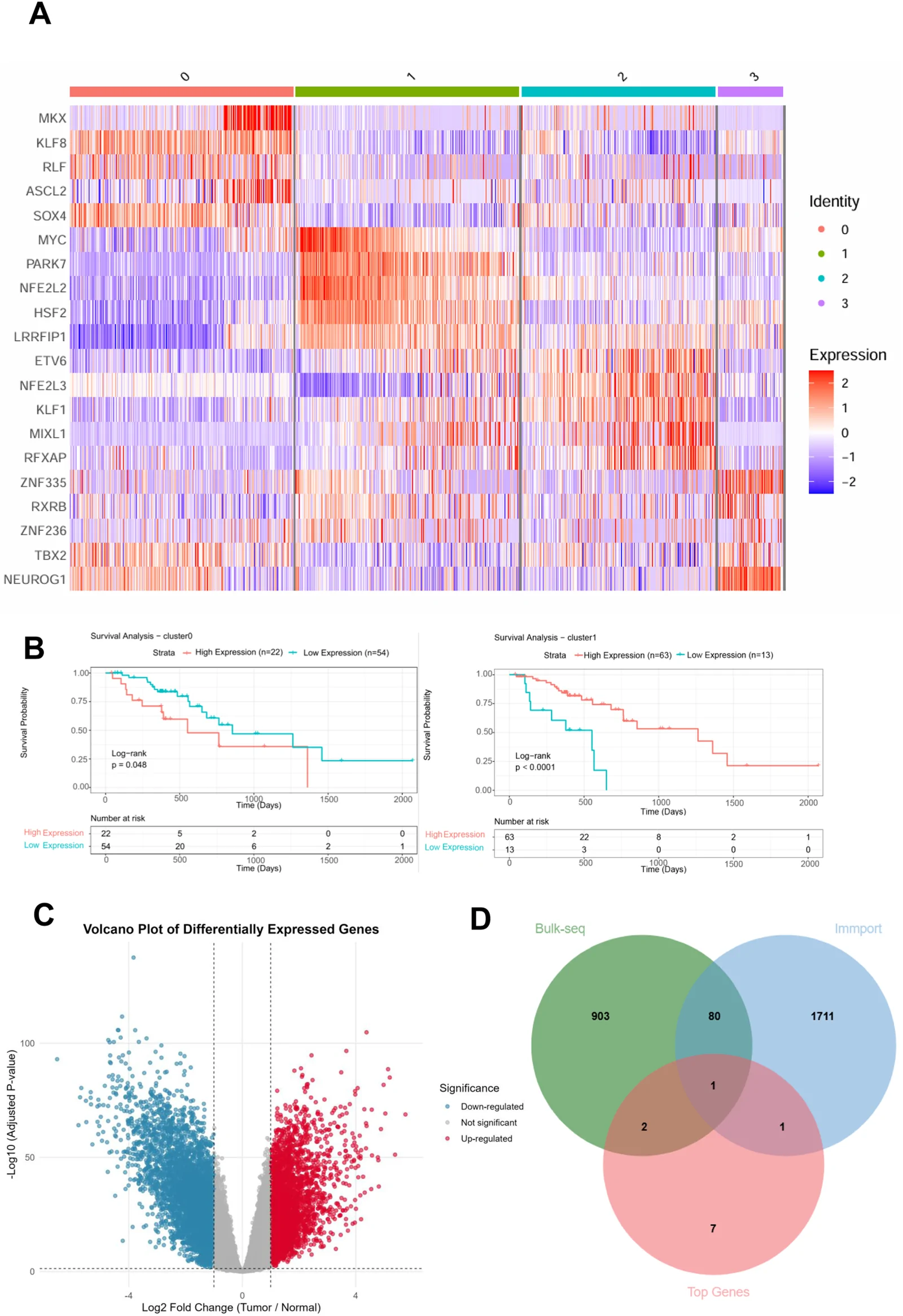
NMB as a Risk Factor for ESCC Metastasis. **(A) **Heatmap of key transcription factor expression across 4 malignant subpopulations. **(B)** Kaplan-Meier survival curves showing that top genes from cluster 0 and cluster 1 are associated with poor prognosis. **(C)** Volcano plot of differentially expressed genes between tumor and normal tissues. **(D)** Venn diagram showing intersections among TCGA cohort, validation cohort, and ImmPort immune gene sets.

Transcription factor expression heatmaps showed regulatory heterogeneity across the 4 malignant subpopulations. Subpopulation 0 expressed stemness and EMT-associated transcription factors ASCL2 and SOX4. Subpopulation 1 expressed proliferation and drug resistance-associated factors MYC and NFE2L2. Subpopulations 2 and 3 displayed lineage differentiation and neuroendocrine-like transcriptional signatures (Figure 3A). Survival analysis based on the top differentially expressed genes from each subpopulation indicated that genes derived from subpopulations 0 and 1 were associated with poor prognosis (Figure 3B; Supplementary Figures S2C, D). Differential expression analysis between tumor and normal tissues in the GSE53624 and TCGA-ESCA cohorts generated a list of upregulated genes. To identify prognosis-associated immune-related genes with potential clinical relevance (Figure 3C; Supplementary Figure S5C), we intersected the top differentially expressed genes from subpopulations 0 and 1 with ESCC-upregulated genes from bulk analysis and immune-related genes from the ImmPort database Intersection of subpopulation 0 and 1 top genes, ESCC upregulated genes, and ImmPort immune-related genes identified NMB as a prognosis-associated immune-related gene (Figure 3D). Malignant cells were stratified into NMBhigh and NMBlow groups by median NMB expression. NFE2 family transcription factors (NFE2, NFE2L1, NFE2L2) were significantly upregulated in NMBhigh cells, indicating enhanced activity of NFE2-mediated transcriptional regulation (Supplementary Figure S5E).

### 3.4 SIDISH Prognostic risk scoring identifies NMB high expression as a risk factor for poor prognosis

**FIGURE 4:**
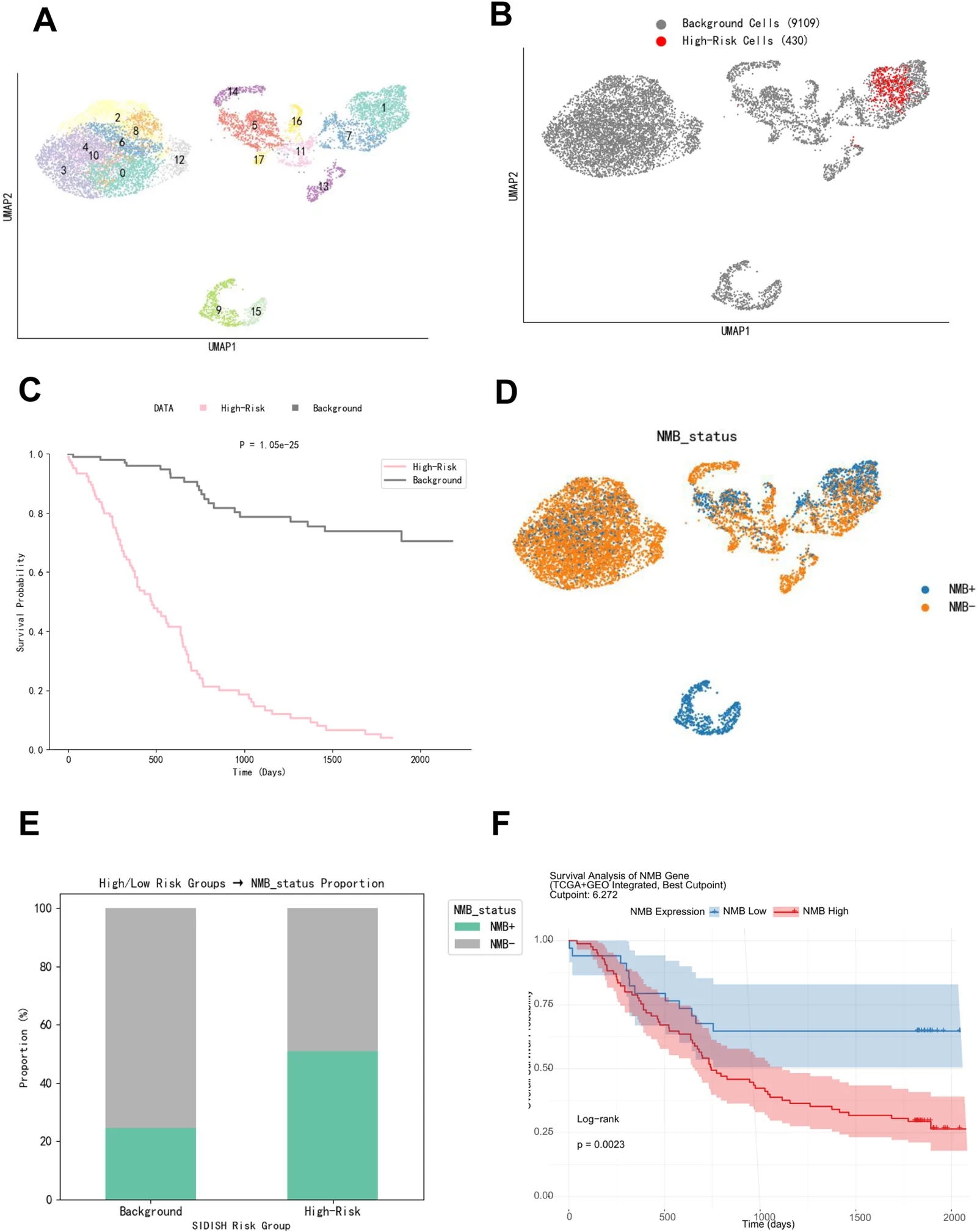
SIDISH Prognostic Risk Scoring Identifies NMB High Expression as a Risk Factor for Poor Prognosis. **(A)** UMAP visualization of all cells showing subpopulation clustering. **(B)** UMAP projection showing distribution of high-risk cells (red, n = 430) and background cells (gray, n = 9109). **(C)** Kaplan-Meier survival analysis comparing high-risk and background groups. **(D)** UMAP showing spatial distribution of NMB-positive and NMB-negative cells. **(E)** Stacked bar chart showing NMB-positive and NMB-negative cell proportions in background and high-risk groups. **(F)** Kaplan-Meier survival curves for NMB-high and NMB-low groups based on optimal cut-off value.

The SIDISH single-cell prognostic risk scoring model assigned 430 cells to the high-risk group and 9,109 cells to the background group (Figures 4A,B). Survival analysis of patients corresponding to high-risk cell gene expression patterns showed shortened survival (HR = 4.12, log-rank *P* < 0.001) (Figure 4C). NMB-positive cells constituted 50% of the high-risk group and 20% of the low-risk group (*P* < 0.01) (Figures 4D,E). Kaplan-Meier analysis confirmed shorter overall survival for patients with high NMB expression (*HR* = 2.98, log-rank *P* = 0.03) (Figure 4F).

### 3.5 NMB high expression correlates with a distinct genomic mutation profile

**FIGURE 5:**
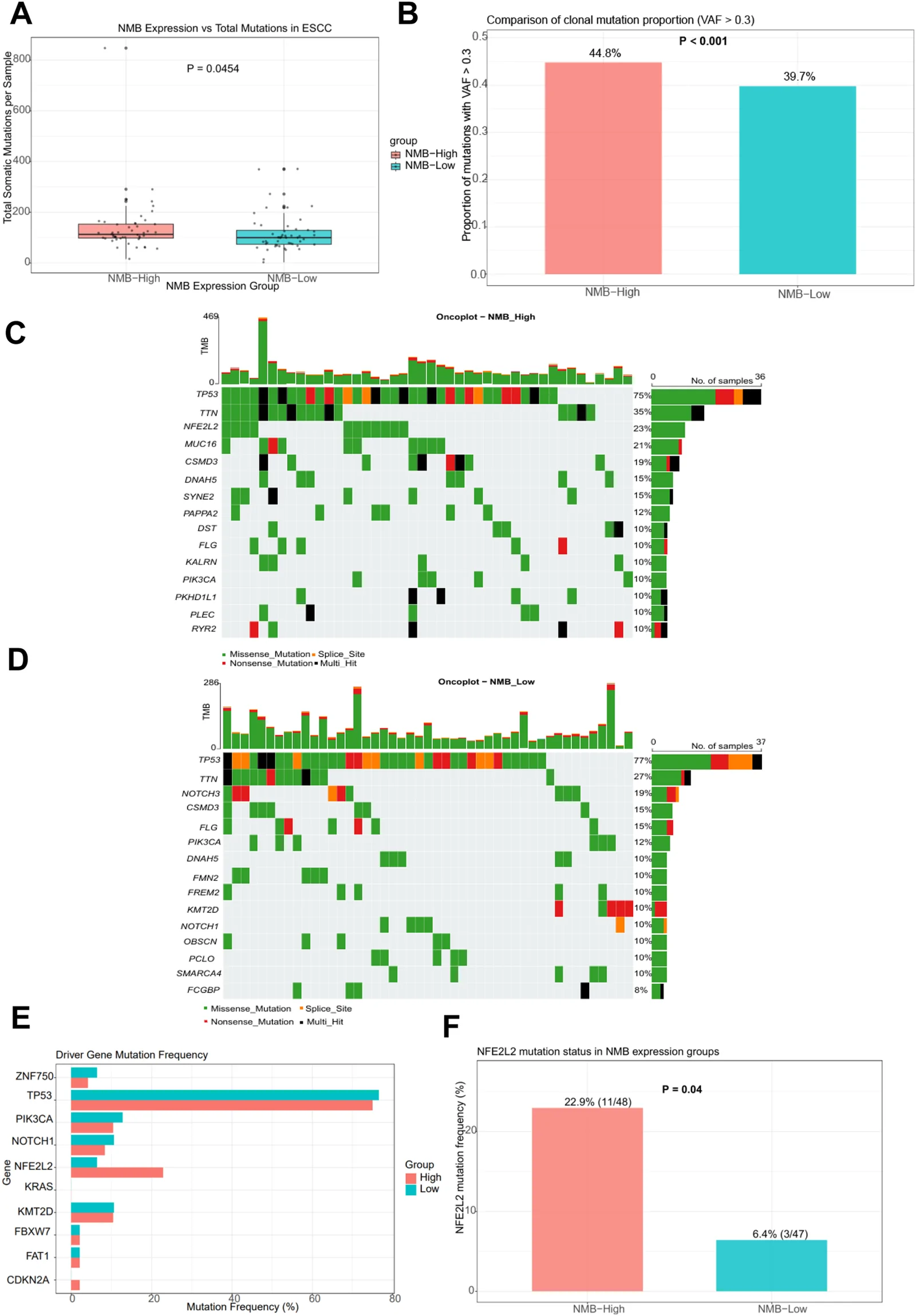
NMB High Expression Correlates with a Distinct Genomic Mutation Profile. **(A)** Box plot of total tumor mutation burden between the two groups. **(B)** Bar chart comparing clonal mutation proportions (VAF > 0.3) between NMB-high and NMB-low groups. **(C,D)** Mutation waterfall plots showing high-frequency driver gene mutations and sample distributions in each group. **(E)** Horizontal bar chart comparing core driver gene mutation frequencies between the two groups. **(F)** Bar chart showing NFE2L2 mutation frequency difference between groups.

TCGA-ESCC samples were divided into NMB-high and NMB-low groups based on median NMB expression. Genome-wide tumor mutation burden was significantly higher in the NMB-high group relative to the low-expression group (*P* = 0.0454)(Figure 5A). Consistently, when applying VAF > 0.3 as the threshold for clonal mutations, the NMB-high group also displayed a markedly increased clonal mutation proportion (44.8% vs. 39.7%, *P* < 0.001) (Figure 5B). TP53 maintained comparable mutation rates in both groups. Beyond TP53, the NMB-high group showed enriched mutations in NFE2L2 (23% vs. 8%, P = 0.04), MUC16 (21%), SYNE2 (15%), and PAPPA2 (12%). The NMB-low group carried mutations in NOTCH3 (19%), FLG (15%), KMT2D (10%), and NOTCH1 (10%)(Figures 5C–F; Supplementary Figures S3A, B). Further stratification of the TCGA-ESCC cohort based on NFE2L2 mutation status indicated that NMB expression was higher in samples carrying NFE2L2 mutations (Supplementary Figure S5D). NFE2L2 encodes a key regulator of the oxidative stress response, and its gain-of-function mutations constitutively activate antioxidant pathways.

### 3.6 NMB-associated functional enrichment and identification of AKR1C1 as a downstream effector

**FIGURE 6:**
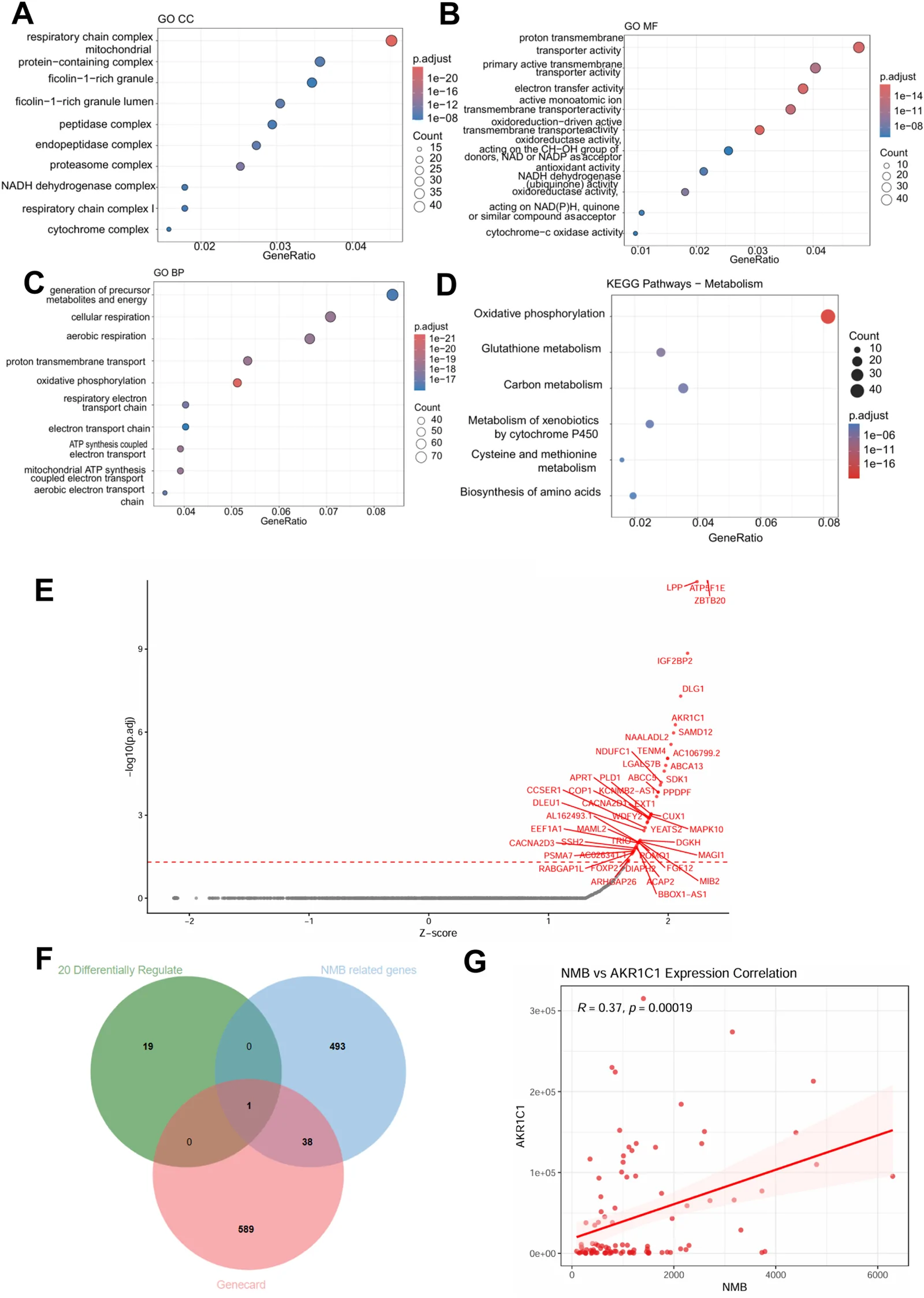
NMB-Associated Functional Enrichment and Identification of AKR1C1 as a Downstream Effector. **(A)** GO cellular component enrichment results. **(B)** GO molecular function enrichment results. **(C)** GO biological process enrichment results. **(D)** KEGG pathway enrichment results. **(E)** Volcano plot of perturbed genes following NMB knockout. **(F)** Venn diagram of differentially regulated genes, NMB-associated genes, and Genecard ROS-related database genes. **(G)** Scatter plot demonstrating the correlation between NMB and AKR1C1 expression (Pearson R = 0.37, *P* = 0.00019).

Differential expression analysis compared NMB-positive and NMB-negative malignant cells using Seurat’s Wilcoxon rank-sum test. Upregulated genes in NMB-positive cells (log2FC > 0.25, expressed in at least 10% of cells) were subjected to KEGG and GO enrichment. GO cellular component enrichment localized the encoded proteins to the mitochondrial respiratory chain and NADH dehydrogenase complexes (Figure 6A). GO molecular function enrichment pointed to oxidoreductase activity, electron transfer, and proton transmembrane transport (Figure 6B). GO biological process enrichment converged on oxidative phosphorylation, mitochondrial respiratory chain electron transport, ATP biosynthesis, and aerobic respiration (Figure 6C). KEGG pathway analysis showed enrichment in oxidative phosphorylation (Figure 6D). To delineate downstream mediators, *in silico* NMB knockout perturbed multiple tumor-associated genes (Figure 6E; Supplementary Figure S4A), with KEGG enrichment pointing to reactive oxygen species metabolism (Supplementary Figure S4B). Given the enrichment of NMB-associated differentially expressed genes in ROS metabolism pathways, we intersected ROS-related genes from GeneCards with NMB-correlated genes from bulk RNA-seq data and identified AKR1C1 as a core downstream effector (Figure 6F). Which was also among the top upregulated genes in metastatic subpopulation 0, suggesting its potential involvement in NMB-driven metastatic progression. Bulk RNA correlation analysis showed a significant positive correlation between NMB and AKR1C1 expression (Figure 6G).

### 3.7 NMB expression and its role in shaping the immunosuppressive tumor microenvironment

**FIGURE 7:**
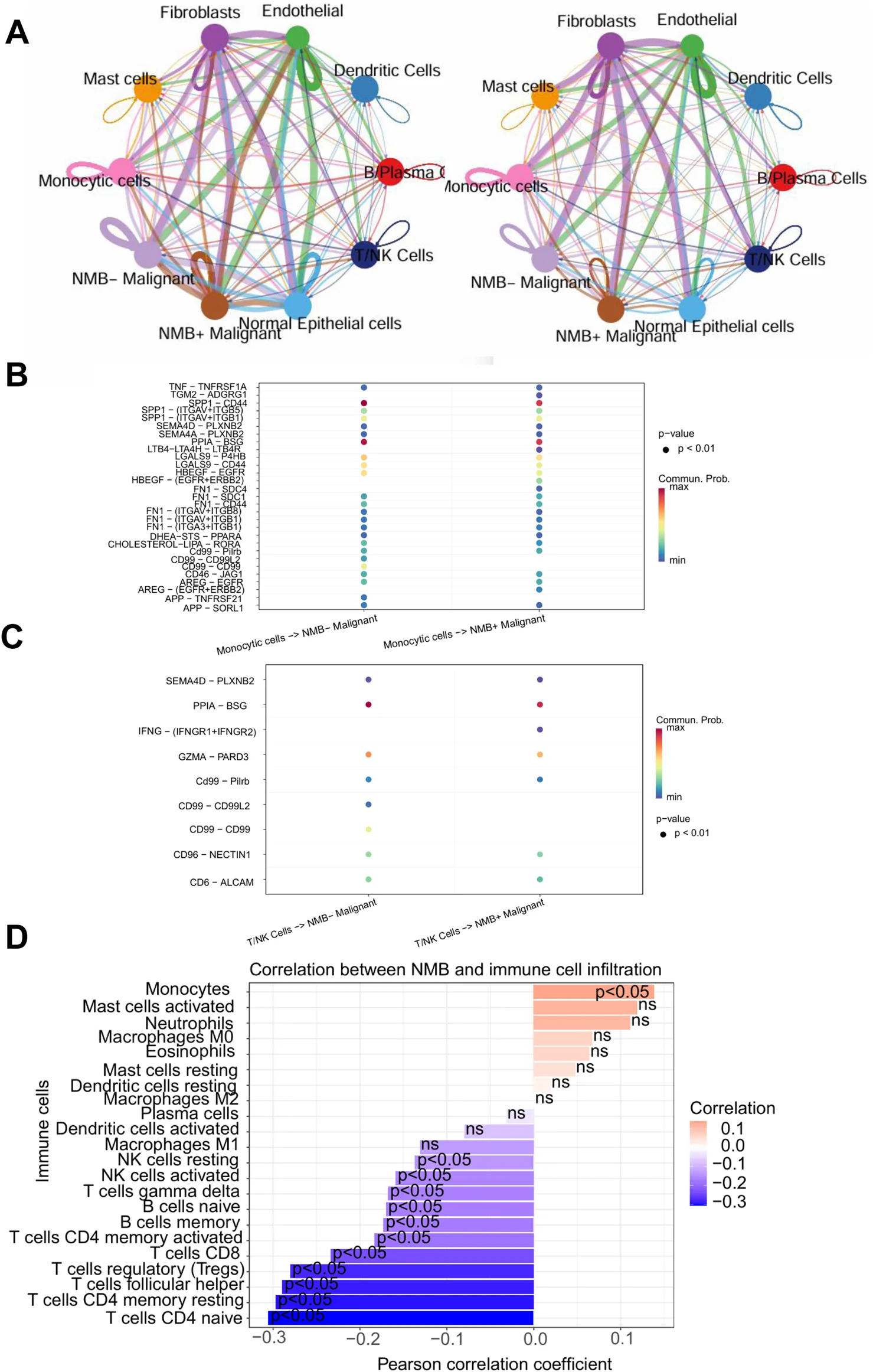
NMB Expression and Its Role in Shaping the Immunosuppressive Tumor Microenvironment. **(A)** Global cell communication network visualization. **(B)** Ligand-receptor interaction bubble plot for monocyte-to-malignant cell signaling. **(C)** Ligand-receptor interaction bubble plot for T/NK cell-to-malignant cell signaling. **(D)** CIBERSORT-based analysis of 22 immune cell types.

To investigate the impact of NMB on intercellular communication within the ESCC microenvironment, we performed CellChat analysis to map receptor-ligand interaction networks (Figure 7A). In both NMB-positive and NMB-negative conditions, monocyte-malignant cell interactions involved inflammatory, adhesion, and growth factor-related pathways. Notably, the SPP1-CD44 and PPIA-BSG pathways were more active in NMB-negative cells, whereas the TGM2-ADGRG1 pathway was enhanced in NMB-positive cells (Figure 7B). T/NK cell-malignant cell interactions engaged PPIA-BSG, SEMA4D-PLXNB2, and CD99 family-related pathways irrespective of NMB status; however, IFNG-(IFNGR1+IFNGR2) signaling was detected exclusively in NMB-positive cells, suggesting a potential role for NMB in modulating antitumor immunity (Figure 7C).

Immune infiltration analysis revealed that NMB expression was negatively correlated with the majority of immune cell types examined, including T cells (CD8^+^, CD4^+^ memory, follicular helper, Tregs), NK cells (resting and activated), B cells (naive, memory, plasma), macrophages (M1, M2), and dendritic cells (resting and activated) (all *P* < 0.05). Conversely, monocyte infiltration showed a positive correlation with NMB expression (*P* < 0.05), whereas M0 macrophages, eosinophils, neutrophils, and activated mast cells exhibited no significant correlations (Figure 7D; Supplementary Figure S4C; Supplementary Figures S5A, B). These findings collectively suggest that NMB expression is closely associated with an immune-desert TME phenotype, marked by pervasive negative correlations with most immune populations and a relative enrichment of monocytes.

### 3.8 CMap screening identifies a potential signaling axis for therapeutic exploration in NMB-Associated ESCC

**FIGURE 8:**
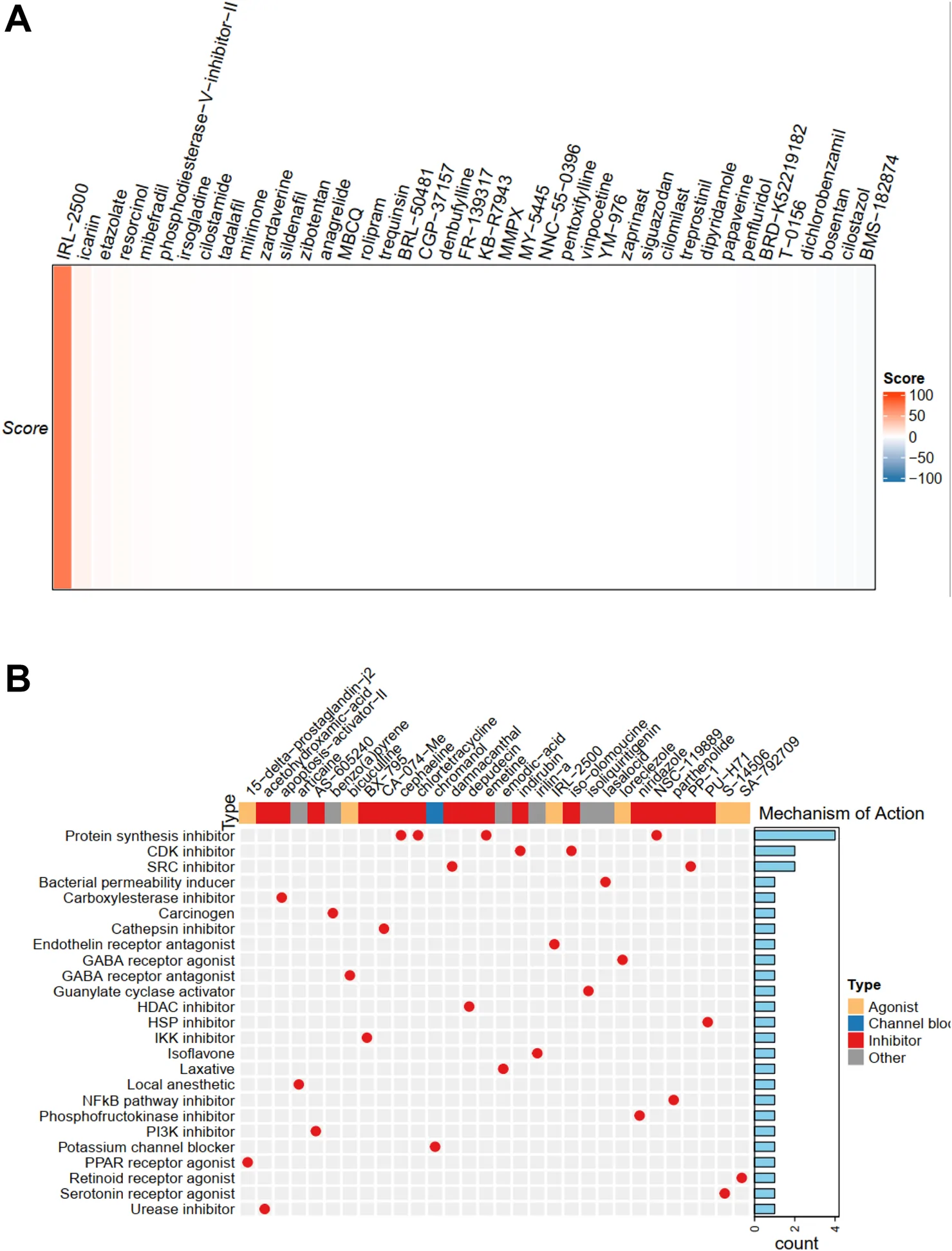
CMap Screening Identifies a Potential Signaling Axis for Therapeutic Exploration in NMB-Associated ESCC. **(A)** Heatmap of candidate drug connectivity scores. **(B)** Mechanism of action (MoA) classification statistics for candidate drugs.

To identify potential therapeutic agents targeting NMB-positive malignant cells, we performed CMap analysis to screen small-molecule compounds capable of reversing their transcriptional profile. Among the candidates, the ETB receptor-selective antagonist IRL-2500 achieved the highest positive connectivity score. Multiple PDE5 inhibitors and vasodilators also showed positive connectivity, whereas bosentan and BMS-182874 exhibited negative scores (Figure 8A). Target enrichment analysis revealed that these candidate compounds converged on the endothelin-PDE5-cGMP signaling axis, calcium transport, and vascular regulation pathways (Figure 8B). These results indicate that the aforementioned pathways participate in NMB-mediated oncogenic effects in ESCC.

### 3.9 NMB depletion suppresses malignant phenotypes in ESCC cell lines TE-1 and KYSE30

**FIGURE 9:**
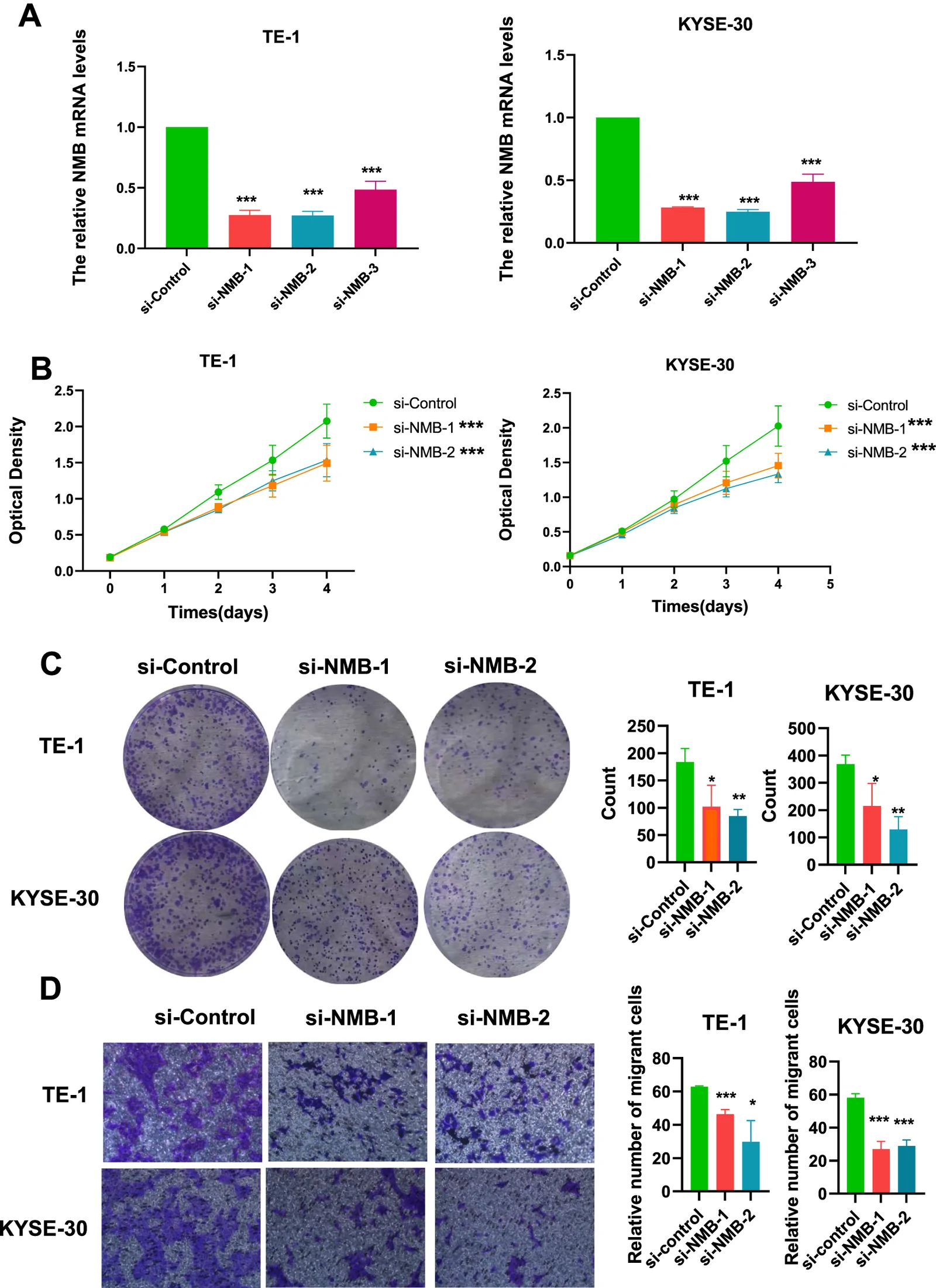
NMB Depletion Suppresses Malignant Phenotypes in ESCC Cell Lines TE-1 and KYSE30. **(A)** qRT-PCR validation of NMB knockdown efficiency. **(B)** MTT proliferation curves over 4 days. **(C)** Colony formation assay staining and quantification. **(D)** Transwell migration assay crystal violet staining and quantification. *P < 0.05, **P < 0.01, ***P < 0.001, ****P < 0.0001.

TE-1 and KYSE30 cells were transfected with three independent NMB-targeting siRNAs. qRT-PCR confirmed that all three siRNAs significantly reduced NMB mRNA expression compared with si-Control (*P* < 0.001)(Figure 9A), with si-NMB-1 and si-NMB-2 exhibiting the most potent silencing efficiency. These two siRNAs were therefore selected for subsequent functional assays. In both cell lines, NMB knockdown significantly inhibited cell proliferation colony formation, and migration, as reflected by reduced proliferation rates, fewer and smaller colonies, and decreased migrated cell numbers (Figures 9B–D). These results indicate that NMB may act as a key functional regulator of proliferation and migration in ESCC cells.

## 4 Discussion

This study identified NMB as a key gene enriched in metastatic ESCC lesions, with its functional signature characterized by driving mitochondrial oxidative phosphorylation and antioxidant detoxification metabolic programs. This gene may confer a selective survival advantage to tumor cells during metastatic colonization by remodeling the immunosuppressive tumor microenvironment.

Previous oncology studies have reported aberrant NMB expression in colorectal cancer, cervical cancer, and glioblastoma, and have established that NMB drives tumor cell proliferation, invasion, and perineural invasion via autocrine and paracrine signaling (Gao et al., 2024; Fan et al., 2025; Li et al., 2023). The transcriptomic profile of this subpopulation revealed high expression of the aldo-keto reductase family members AKR1C1, AKR1C2, and AKR1B10. These enzymes function primarily to eliminate lipid peroxides and maintain intracellular redox homeostasis (Su et al., 2026; Song et al., 2020). Cell migration and cytoskeletal remodeling demand substantial energy supply, and oxidative phosphorylation serves as the main source of ATP (Wu et al., 2021). KEGG and GO enrichment analyses revealed that upregulated genes in NMB-positive cells were mainly enriched in oxidative phosphorylation, mitochondrial respiratory chain complexes and ATP biosynthesis pathways. *In vitro* NMB knockdown markedly inhibited the proliferation and migration of tumor cells, indicating that NMB is essential for maintaining cellular metabolic homeostasis. In silico perturbation analysis identified AKR1C1 as a core downstream effector of NMB. The expression of AKR1C1 is positively regulated by NMB, and its encoded protein maintains intracellular redox balance and mitigates metabolic stress induced by intensified mitochondrial respiration. The coordination of energy production and redox regulation confers a survival advantage on metastatic tumor subclones and facilitates their adaptation to the microenvironment during distant colonization.

Genomic analysis provided additional mechanistic support. Mitochondrial ROS generated during sustained oxidative phosphorylation is a known source of DNA damage (Sabharwal and Schumacker, 2014). Tumors with high NMB expression show higher clonal mutation fractions and tumor mutational burden, indicating oxidative stress induces genomic instability. Mutational patterns differ greatly between NMB-high and NMB-low tumors. Activating missense mutations of NFE2L2, which encodes the core antioxidant transcription factor NRF2, are more prevalent in NMB-high tumors. These mutations target the Neh2 domain, block KEAP1-dependent NRF2 degradation and lead to persistent NRF2 activation (Takahashi et al., 2024). Based on previous reports that the AKR1C1 promoter contains AREs and is regulated by NRF2 (Su et al., 2020). It is plausible that NRF2 activation contributes to AKR1C1 upregulation in NMB-high tumors, although this remains a speculative interpretation requiring experimental validation.

The immune microenvironment also distinguished NMB-positive from NMB-negative cells. NMB expression correlated negatively with CD8^+^ T cell and NK cell infiltration, and positively with monocyte abundance. CellChat revealed active signaling between monocytes and NMB-positive cells via the TGM2–ADGRG1 pair. Tumor-secreted TGM2 can polarize monocytes toward M2-like macrophages, while ADGRG1 mediates adhesion and migration. This pathway may enable NMB-positive cells to recruit monocytes and promote their differentiation into tumor-associated macrophages, fostering immunosuppression. Additionally, IFNG signaling was detected only between NMB-positive cells and T/NK cells. Although interferon-γ is cytotoxic, chronic exposure can induce PD-L1 on tumor cells (Liu, 2025), potentially driving adaptive immune resistance. Whether NMB-positive cells use this to evade T-cell clearance requires experimental testing, but the correlation and ligand–receptor data support this possibility. Connectivity Map screening identified the endothelin–PDE5–cGMP axis as a therapeutic pathway in NMB-positive ESCC. This axis regulates vascular tone and calcium flux, and PDE5 inhibitors sustain antitumor immunity by restoring dendritic cell motility (Tang et al., 2025); consistent with the immune-desert phenotype in NMB-high tumors. However, these predictions await preclinical validation, and the efficacy of such agents against NMB-driven tumors remains unclear. Overall, our results position NMB as a driver of metastatic adaptation via metabolic, genomic, and immune remodeling, and provide a rationale for evaluating its downstream effectors as therapeutic targets in metastatic ESCC.

Our analysis of the metastatic microenvironment is based solely on lung metastatic samples and has not included other metastatic sites; thus, the broader applicability of our findings needs to be examined in future studies incorporating multi-site data. The direct mechanism by which NMB regulates mitochondrial respiratory chain activity or AKR1C1 transcription remains unknown and warrants further study. CMAP-predicted compounds have not been tested in animal models, and their efficacy against NMB-positive tumors is unclear. Nonetheless, our findings position NMB as a key driver of metastatic adaptation via coordinated metabolic, genomic, and immune remodeling, and provide a rationale for evaluating its downstream effectors as therapeutic targets in metastatic esophageal squamous cell carcinoma.

## Data Availability

Publicly available datasets were analyzed in this study. These data can be accessed here: [TCGA database (https://portal.gdc.cancer.gov/) and GEO database (https://www.ncbi.nlm.nih.gov/geo/query/acc.cgi, accession numbers: GSE309392, and GSE53624)].

## Supplementary material

SUPPLEMENTARY FIGURE S1 Single-cell transcriptomic landscape of primary and metastatic ESCC samples. **(A,B)** UMAP visualization of all cells colored by **(A)** patient ID (P1–P6) and **(B)** sample origin (primary vs. metastatic lesions).**(C,D)** UMAP plots showing the annotation of major cell types based on canonical marker genes. Cells are colored by **(C)** cell type and **(D)** grouped by sample origin.**(E,F)** Re-clustering of epithelial cells from all samples. **(E)** Epithelial cell subclusters colored by cluster ID. **(F)** Epithelial cells colored by sample origin (primary vs. metastatic).**(G)** InferCNV-based classification of epithelial cells into malignant (red) and normal (blue) cells, based on inferred copy number variations.**(H)** UMAP visualization of the final cell-type annotation for all cells in the data.

SUPPLEMENTARY FIGURE S2 Subclustering and prognostic analysis of malignant cells in ESCC. **(A)** UMAP visualization showing the initial subclustering of all malignant epithelial cells (clusters 1–8).**(B)** UMAP plot showing the refined subclustering of malignant cells (clusters 0–3).**(C,D)** Kaplan–Meier survival curves comparing overall survival between patients with high and low expression of signature genes from **(C)** cluster 2 and **(D)** cluster 3, based on the TCGA-ESCC cohort. Log-rank test p-values are indicated.

SUPPLEMENTARY FIGURE S3 Genomic mutational landscape of ESCC tumors stratified by NMB expression. **(A,B)** Summary of somatic mutations in NMB-high **(A)** and NMB-low **(B)** ESCC tumors. The panels include: variant classification (left), variant type (middle left), SNV class (middle right), variants per sample (bottom left), variant classification summary (bottom middle), and the top 10 most frequently mutated genes (bottom right).

SUPPLEMENTARY FIGURE S4 Functional enrichment and immune infiltration analysis of NMB-associated genes. **(A)** Bar plot showing the top 20 upregulated differentially expressed genes (DEGs) after NMB knockdown, ranked by fold change (FC). **(B)** KEGG pathway enrichment analysis of DEGs following NMB knockdown. The dot plot displays the enriched pathway “Chemical carcinogenesis - reactive oxygen species”, with gene ratio on the x-axis and adjusted p-value (p.adjust) indicated by color intensity. **(C)** Pearson correlation analysis between NMB expression and immune cell infiltration estimated by xCell. The bar plot shows the correlation coefficients (R) between NMB and different immune cell types, colored by correlation strength (red: positive correlation; blue: negative correlation).

SUPPLEMENTARY FIGURE S5
**(A)** Pearson correlation between NMB expression and MCP-counter immune cell scores. Red indicates positive correlations, blue indicates negative correlations. **(B)** Pearson correlation between NMB expression and ESTIMATE scores (ImmuneScore, StromalScore, ESTIMATEScore). Blue bars indicate negative correlations. **(C)** NMB expression in tumor vs. normal tissues in the integrated cohort. NMB expression (ComBat-corrected, log2 scale) was significantly higher in tumor tissues than in normal tissues (p > 2e-16). **(D)** NMB expression by NFE2L2 mutation status in ESCC. Mutant tumors showed significantly higher NMB expression than wild-type tumors (p > 0.001). **(E)** The heatmap presents top TFs with differential activity between high and low NMB expression groups. Color intensity represents regulatory activity (red: higher; blue: lower). **(F)** UMAP and t-SNE clustering of cells before and after integration using the Harmony algorithm. **(G)** Cellular distribution visualized by UMAP and t-SNE before and after Harmony batch effect removal.
