# Supplementary material for: Integrative single-cell and genomic analysis reveals NMB as a driver of metastatic adaptation in esophageal squamous cell carcinoma via metabolic rewiring and immune evasion
Source: Front Cell Dev Biol. 2026 Aug 31;14:1914292. doi: 10.3389/fcell.2026.1914292 (PMC13572137; doi:10.3389/fcell.2026.1914292)
Supplement: Supplementary file 1 [file DataSheet1.pdf]

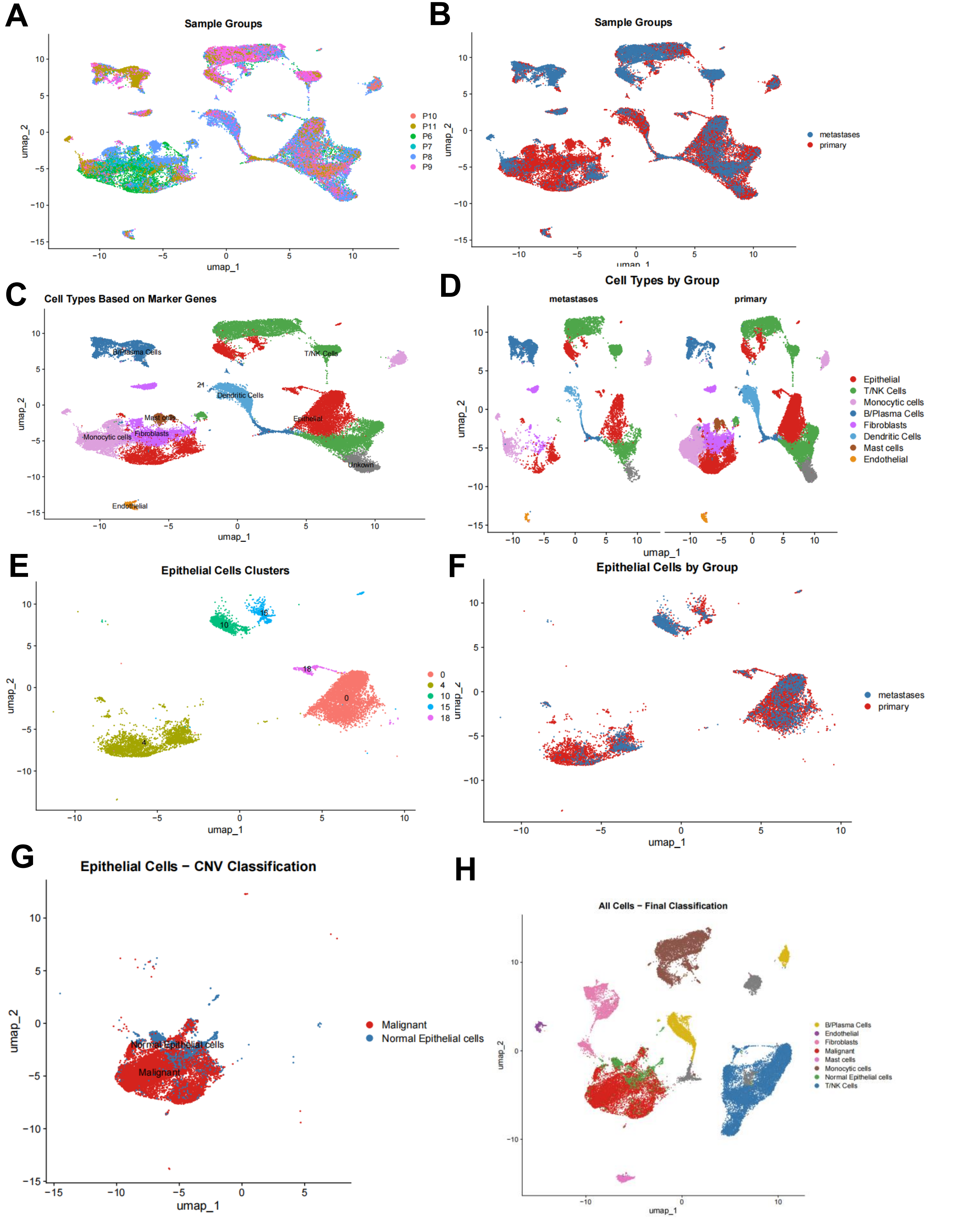
A
Sample Groups
P10
P11
P6
P7
P8
P9
umap_1
umap_2
B
Sample Groups
metastases
primary
C
Cell Types Based on Marker Genes
B/Plasma Cells
T/NK Cells
Dendritic Cells
Mast cells
Monocytic cells
Fibroblasts
Epithelial
Unkown
Endothelial
D
Cell Types by Group
metastases
primary
Epithelial
T/NK Cells
Monocytic cells
B/Plasma Cells
Fibroblasts
Dendritic Cells
Mast cells
Endothelial
E
Epithelial Cells Clusters
0
4
10
15
18
F
Epithelial Cells by Group
metastases
primary
G
Epithelial Cells − CNV Classification
Malignant
Normal Epithelial cells
H
All Cells − Final Classification
B/Plasma Cells
Endothelial
Fibroblasts
Malignant
Mast cells
Monocytic cells
Normal Epithelial cells
T/NK Cells

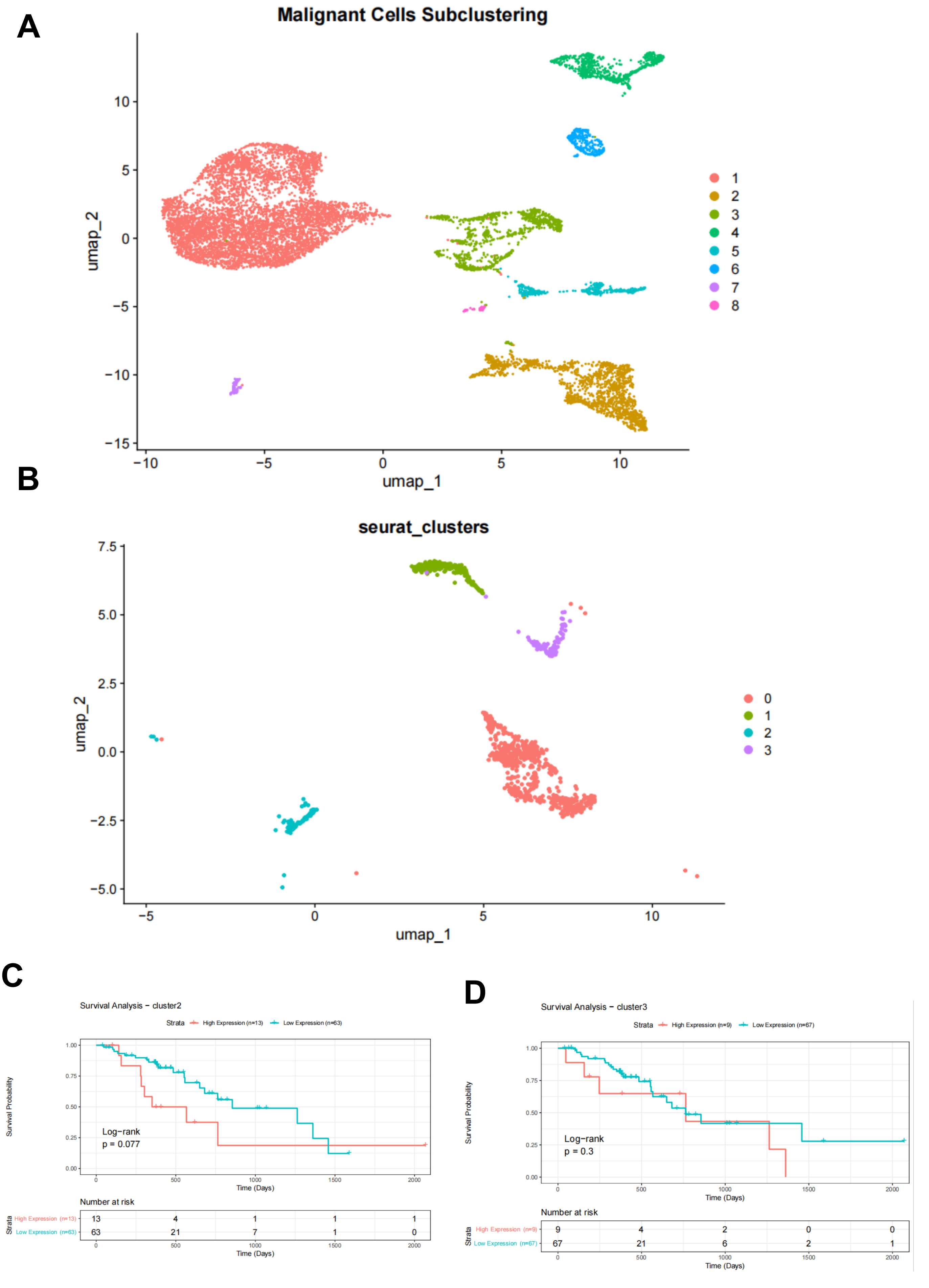
A
Malignant Cells Subclustering
umap_2
umap_1
1
2
3
4
5
6
7
8
B
seurat_clusters
umap_2
umap_1
0
1
2
3
C
Survival Analysis - cluster2
Strata
High Expression (n=13)
Low Expression (n=63)
Survival Probability
Log-rank
p = 0.077
Time (Days)
Number at risk
13 4 1 1 1
63 21 7 1 0
D
Survival Analysis - cluster3
Strata
High Expression (n=9)
Low Expression (n=67)
Survival Probability
Log-rank
p = 0.3
Time (Days)
Number at risk
9 4 2 0 0
67 21 6 2 1

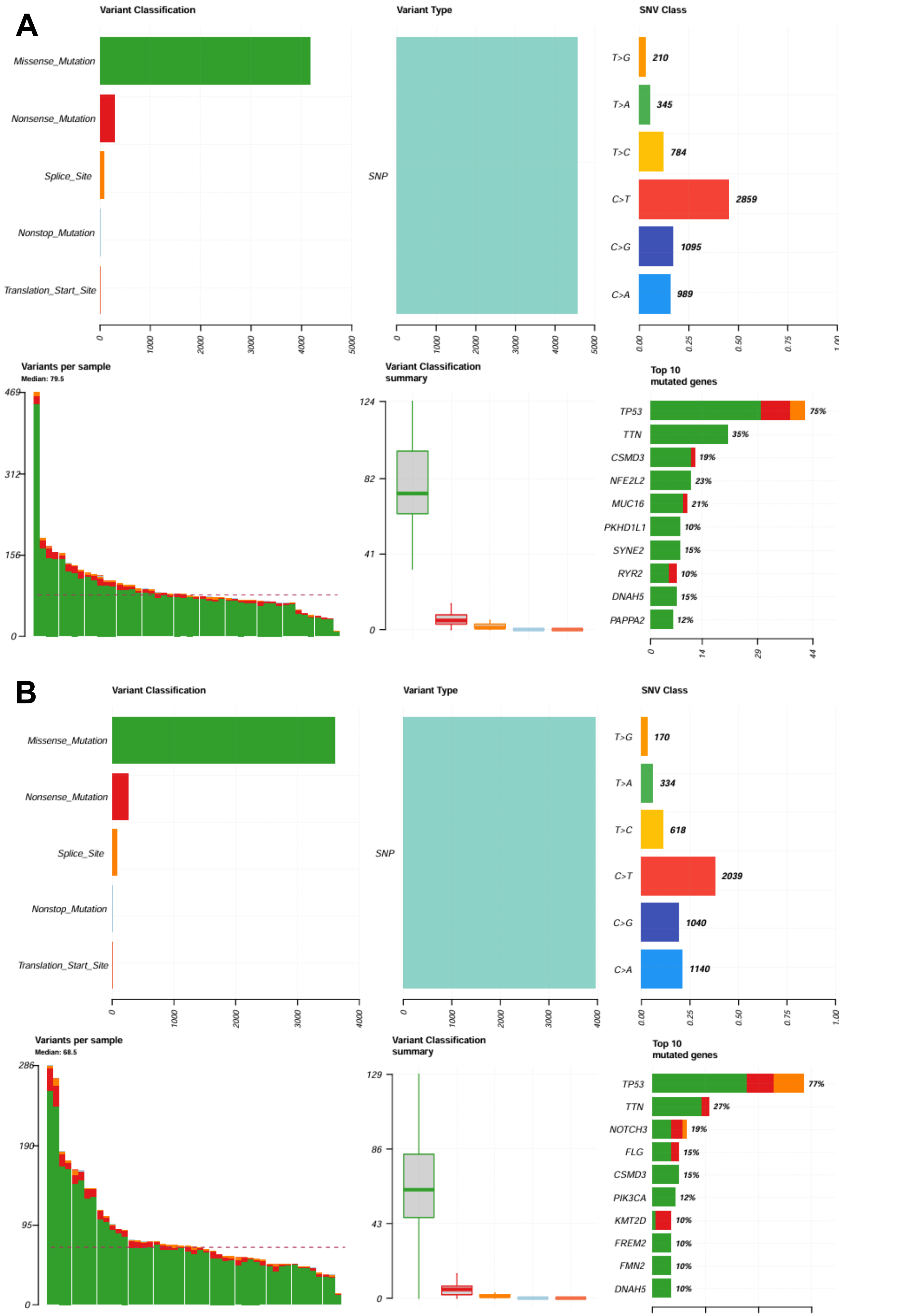
A
Variant Classification
Missense_Mutation
Nonsense_Mutation
Splice_Site
Nonstop_Mutation
Translation_Start_Site
0 1000 2000 3000 4000 5000
Variant Type
SNP
0 1000 2000 3000 4000 5000
SNV Class
T>G 210
T>A 345
T>C 784
C>T 2859
C>G 1095
C>A 989
0.00 0.25 0.50 0.75 1.00
Variants per sample
Median: 79.5
469
312
156
0
Variant Classification summary
124
82
41
0
Top 10 mutated genes
TP53 75%
TTN 35%
CSMD3 19%
NFE2L2 23%
MUC16 21%
PKHD1L1 10%
SYNE2 15%
RYR2 10%
DNAH5 15%
PAPPA2 12%
0 14 29 44
B
Variant Classification
Missense_Mutation
Nonsense_Mutation
Splice_Site
Nonstop_Mutation
Translation_Start_Site
0 1000 2000 3000 4000
Variant Type
SNP
0 1000 2000 3000 4000
SNV Class
T>G 170
T>A 334
T>C 618
C>T 2039
C>G 1040
C>A 1140
0.00 0.25 0.50 0.75 1.00
Variants per sample
Median: 68.5
286
190
95
0
Variant Classification summary
129
86
43
0
Top 10 mutated genes
TP53 77%
TTN 27%
NOTCH3 19%
FLG 15%
CSMD3 15%
PIK3CA 12%
KMT2D 10%
FREM2 10%
FMN2 10%
DNAH5 10%

A

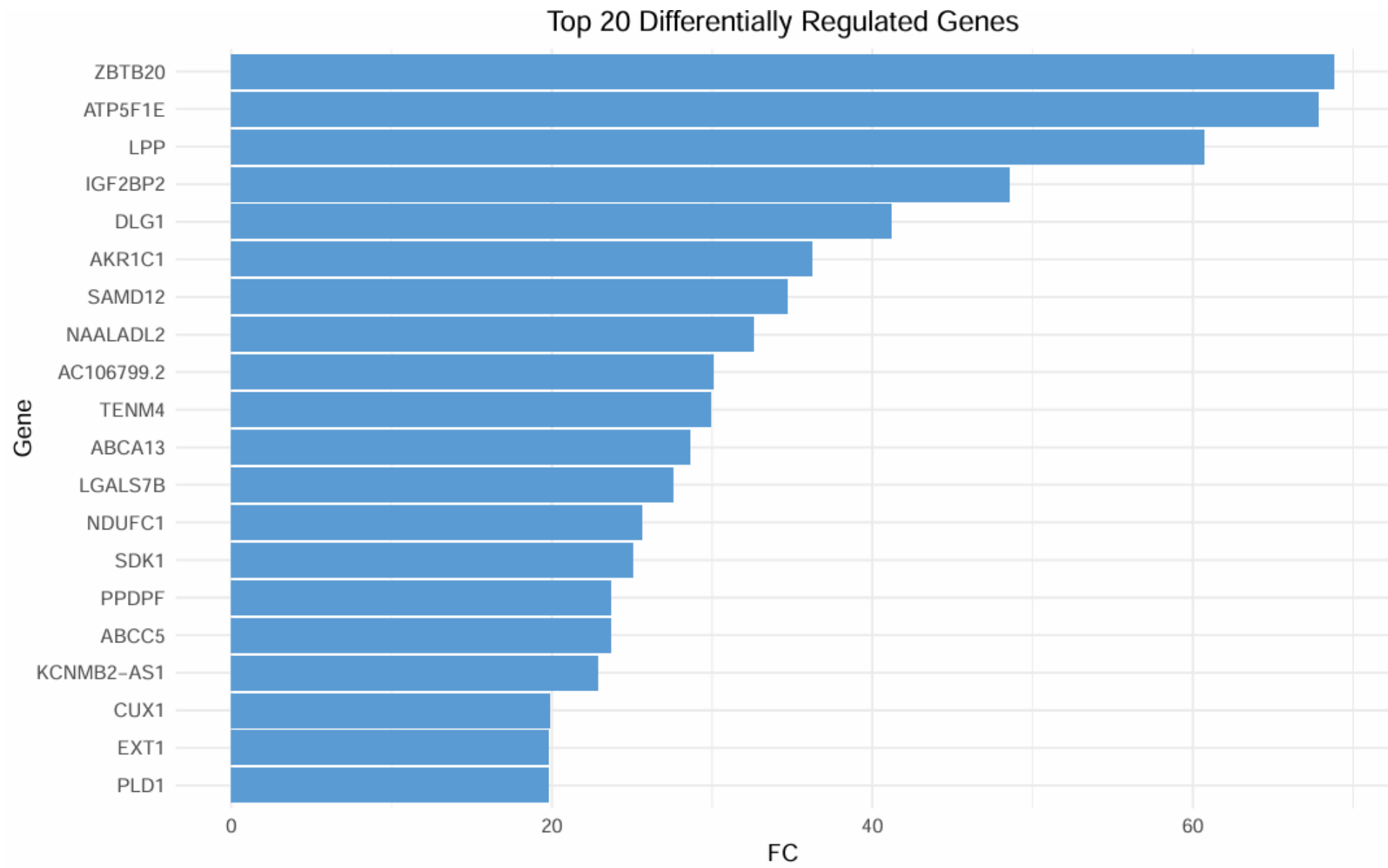

Top 20 Differentially Regulated Genes
ZBTB20
ATP5F1E
LPP
IGF2BP2
DLG1
AKR1C1
SAMD12
NAALADL2
AC106799.2
TENM4
ABCA13
LGALS7B
NDUFC1
SDK1
PPDPF
ABCC5
KCNMB2-AS1
CUX1
EXT1
PLD1
Gene
0
20
40
60
FC

B

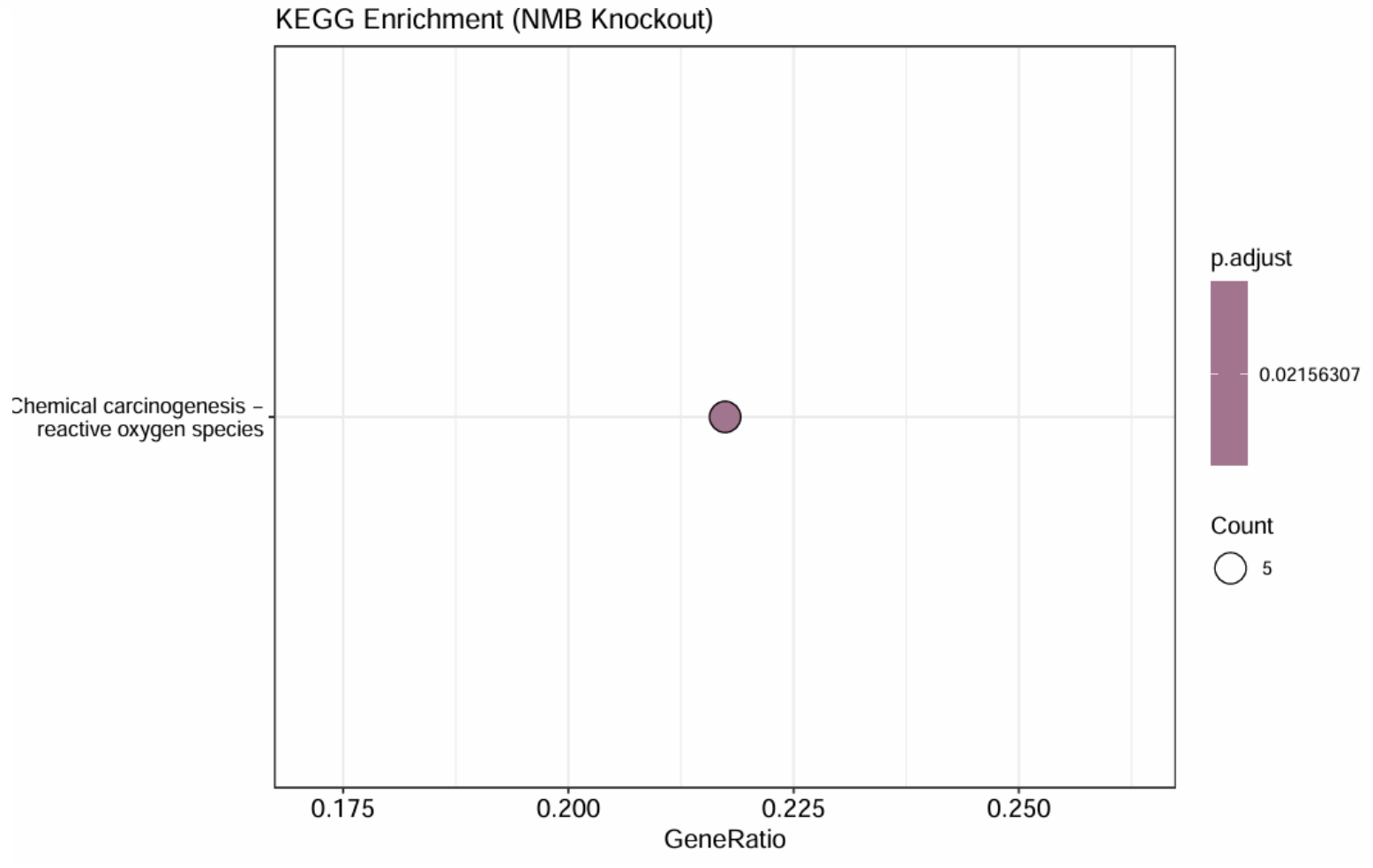

KEGG Enrichment (NMB Knockout)
Chemical carcinogenesis – reactive oxygen species
p.adjust
0.02156307
Count
5
0.175
0.200
0.225
0.250
GeneRatio

C

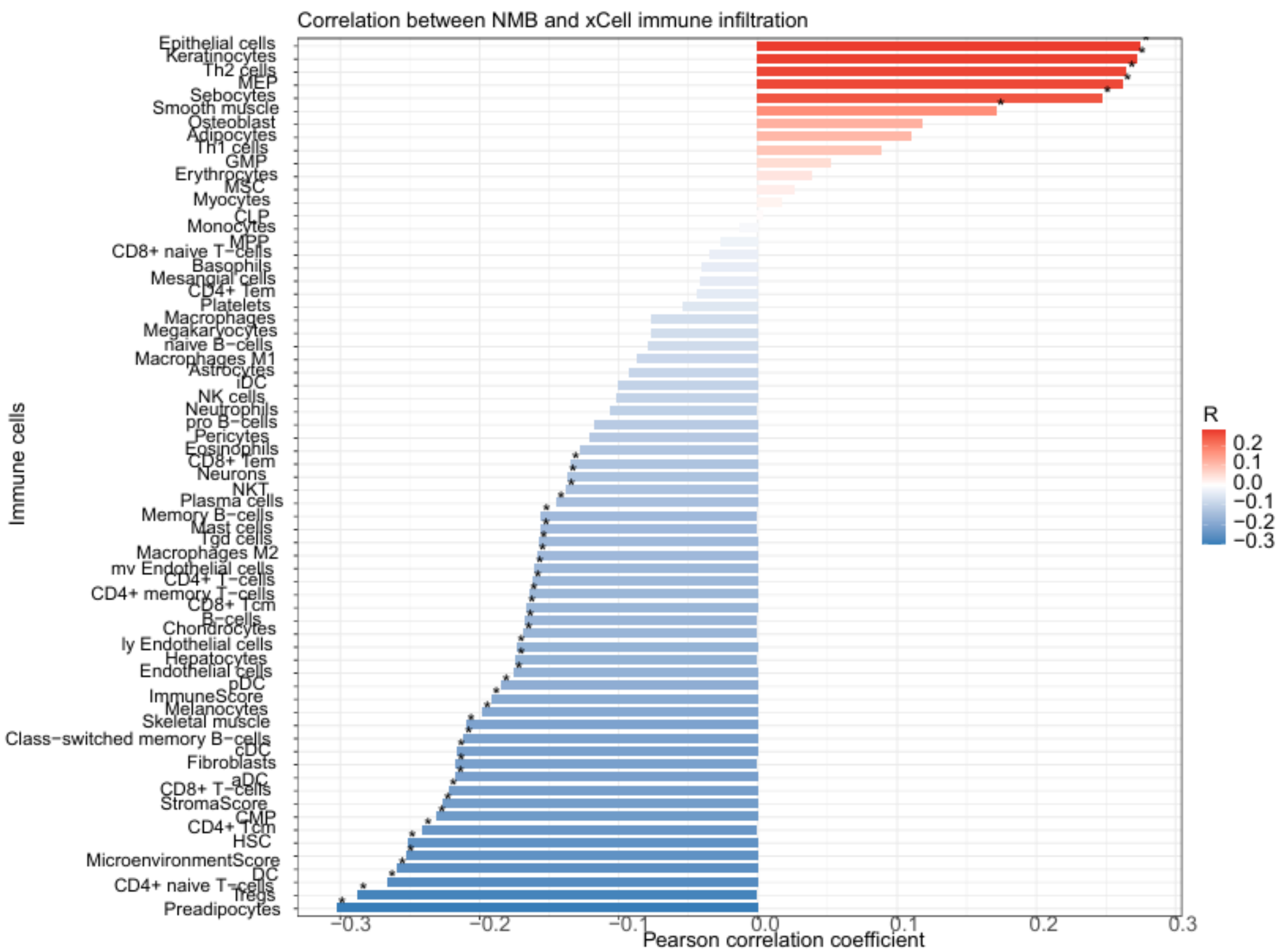

Correlation between NMB and xCell immune infiltration
Epithelial cells
Keratinocytes
Th2 cells
MEP
Sebocytes
Smooth muscle
Osteoblast
Adipocytes
Th1 cells
GMP
Erythrocytes
MSC
Myocytes
CLP
Monocytes
MPP
CD8+ naive T-cells
Basophils
Mesangial cells
CD4+ Tem
Platelets
Macrophages
Megakaryocytes
naive B-cells
Macrophages M1
Astrocytes
iDC
NK cells
Neutrophils
pro B-cells
Pericytes
Eosinophils
CD8+ Tem
Neurons
NKT
Plasma cells
Memory B-cells
Mast cells
Tgd cells
Macrophages M2
mv Endothelial cells
CD4+ T-cells
CD4+ memory T-cells
CD8+ Tcm
B-cells
Chondrocytes
ly Endothelial cells
Hepatocytes
Endothelial cells
pDC
ImmuneScore
Melanocytes
Skeletal muscle
Class-switched memory B-cells
cDC
Fibroblasts
aDC
CD8+ T-cells
StromaScore
CMP
CD4+ Tcm
HSC
MicroenvironmentScore
DC
CD4+ naive T-cells
Tregs
Preadipocytes
Immune cells
−0.3
−0.2
−0.1
0.0
0.1
0.2
0.3
Pearson correlation coefficient
R
0.2
0.1
0.0
−0.1
−0.2
−0.3

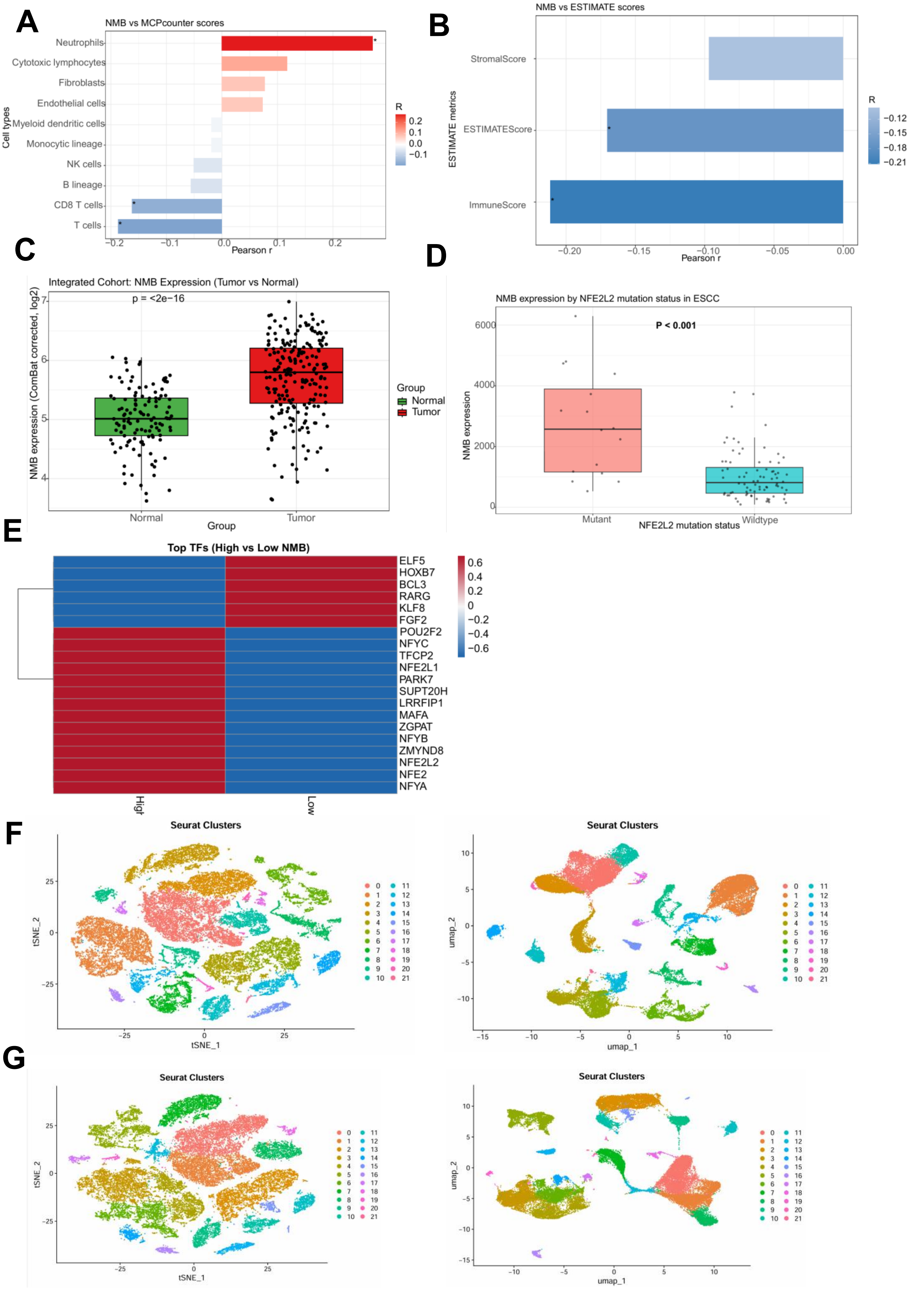

A
NMB vs MCPcounter scores
Neutrophils
Cytotoxic lymphocytes
Fibroblasts
Endothelial cells
Myeloid dendritic cells
Monocytic lineage
NK cells
B lineage
CD8 T cells
T cells
Cell types
Pearson r
R
B
NMB vs ESTIMATE scores
StromalScore
ESTIMATEScore
ImmuneScore
ESTIMATE metrics
Pearson r
R
C
Integrated Cohort: NMB Expression (Tumor vs Normal)
p = <2e−16
NMB expression (ComBat corrected, log2)
Group
Normal
Tumor
D
NMB expression by NFE2L2 mutation status in ESCC
P < 0.001
NMB expression
Mutant
Wildtype
NFE2L2 mutation status
E
Top TFs (High vs Low NMB)
ELF5
HOXB7
BCL3
RARG
KLF8
FGF2
POU2F2
NFYC
TFCP2
NFE2L1
PARK7
SUPT20H
LRRFIP1
MAFA
ZGPAT
NFYB
ZMYND8
NFE2L2
NFE2
NFYA
High
Low
F
Seurat Clusters
tSNE_1
tSNE_2
umap_1
umap_2
G
Seurat Clusters
tSNE_1
tSNE_2
umap_1
umap_2
