# Supplementary material for: Integrative single-cell and genomic analysis reveals NMB as a driver of metastatic adaptation in esophageal squamous cell carcinoma via metabolic rewiring and immune evasion
Source: Front Cell Dev Biol. 2026 Aug 31;14:1914292. doi: 10.3389/fcell.2026.1914292 (PMC13572137; doi:10.3389/fcell.2026.1914292)
Supplement: Supplementary file 2 [file Table1.docx]

Supplementary file

Table S1. The sequences of primers of NMB and GAPDH.

| NMB-Forward Primer | CGCACCCCAAATCCAGTACA |
| --- | --- |
| NMB-Reverse Primer | TCAACAGGGTCCCATTCAGC |
| GAPDH-Forward Primer | GATTCCACCCATGGCAAATTC |
| GAPDH- Reverse Primer | CTGGAAGATGGTGATGGGATT |

Table S2. Oligonucleotides of siRNAs.

| siNMB-1 | sense | GAAUCCUCCUGCUAAAGAA |
| --- | --- | --- |
|  | antisense | UUCUUUAGCAGGAGGAUUC |
| siNMB-2 | sense | GAGGCUGCUGGUACAAAUA |
|  | antisense | UAUUUGUACCAGCAGCCUC |
| siNMB-3 | sense | GUCACUUCAUGGGCAAGAA |
|  | antisense | UUCUUGCCCAUGAAGUGAC |
